# Genome Sequences of the Human-Skin-Originated Brevundimonas albigilva TT17 and the Soil-Originated B. albigilva KEME 9005-016^T^ 

**DOI:** 10.1128/mra.00776-22

**Published:** 2022-09-26

**Authors:** Haelim Son, Seung-Yoon Oh, Kyoung Lee

**Affiliations:** a Department of Bio Health Science, Changwon National University, Changwon, Republic of Korea; b Department of Biology and Chemistry, Changwon National University, Changwon, Republic of Korea; Montana State University

## Abstract

Brevundimonas albigilva TT17 was isolated from human skin. This strain can grow on Triton X-100. Here, we present the whole-genome sequences of this strain and B. albigilva KEME 9005-016^T^. This result demonstrates the first information on the genome sequences of the species of B. albigilva.

## ANNOUNCEMENT

The genus Brevundimonas belonging to the family *Caulobacteraceae* comprises Gram-negative, nonfermenting, and aerobic bacilli (1). B. albigilva KEME 9005-016^T^ with high catalase activity and short wavelength flagella was isolated from forest soil in South Korea (2). We report here the genome sequences of the species of B. albigilva with strains of TT17 and KEME 9005-016^T^. The comparative genomes will provide information on genetic traits for strain adaptation to the different niches.

A swab sample from the forehead skin of a male college student was directly streaked on minimal salts basal (MSB) medium agar (3) containing 0.5% Triton X-100 as a source of carbon and energy. The agar medium was aerobically incubated for 1 week at 28°C for growth. One isolate, named TT17, was further purified by streaking on nutrient broth agar (Difco Co.) with 0.1% sodium pyruvate following incubation at 28°C for 2 days. This strain was deposited in the Korean Collection for Type Cultures as KCTC 92370. Ethical approval for subject sampling was granted by the institutional review board of Changwon National University. Sanger sequencing of the 16S rRNA gene (4) using the V1 to V9 region (i.e., with primers 27F and 1492R) enabled the identification of TT17 as belonging to the genus Brevundimonas.

For DNA extraction, cells were cultured in a flask on nutrient broth with 0.1% sodium pyruvate for 48 h at 28°C with shaking at 140 rpm. Total genomic DNA was purified using the phenol extraction method (5). Genomic DNA was sequenced with Illumina and Oxford Nanopore Technologies MinION platforms. Illumina sequencing was performed at DNALink Co. (Seoul, Republic of Korea). The whole-genome sequencing was performed using a TruSeq DNA PCR-free 550-bp library kit (Illumina) and demultiplexing by bcl2fastaq2 (version 2.20) on the Illumina NovaSeq6000 sequencer. The quality of the raw sequencing data were checked using FastQC with ASCII Q score offset 33 (https://www.bioinformatics.babraham.ac.uk/projects/fastqc/) (6). The read length was 2 × 151 of approximately 550-bp insert size, and the mean Phred quality score was 35.54. For Nanopore sequencing, libraries were prepared with the SQK-LSK109 kit and multiplexed using the EXP-NBD104 barcoding kit according to the protocol. Sequencing was performed on a MinION sequencer (v20.10.3) using an R9.4.1 flow cell with default settings. Reads were base called and demultiplexed using Guppy v3.4.1 in high accuracy mode with the minimum Q score of 8. For comparison, B. albigilva KEME 9005-016^T^ obtained from the Korean Agricultural Culture Collection was cultivated, and genomic DNA was purified under the same conditions described for TT17. The genomic DNA of KEME 9005-016^T^ was sequenced with the Nanopore platform. The reads of strains TT17 and KEME 9005-016^T^ were assembled *de novo* using Unicycler version 0.4.9 and Miniasm version 0.3 with default settings, respectively (7). The quality of the assembled genome sequences was evaluated using CheckM version 1.1.3 (8). The protein sequence-based comparison between genomes was analyzed using Rapid Annotations using Subsystems Technology (RAST) Seed Viewer (9). The cumulative GC skew and GC content were depicted using the CGview program with sliding window sizes of 1,000- and 100-bp steps (10). The default parameters were used for all software unless otherwise noted. Gene predictions and annotations were provided by NCBI using the Best-placed reference protein set; GeneMarkS-2+ of the NCBI Prokaryotic Genome Annotation Pipeline 6.1 (11).

**FIG 1:**
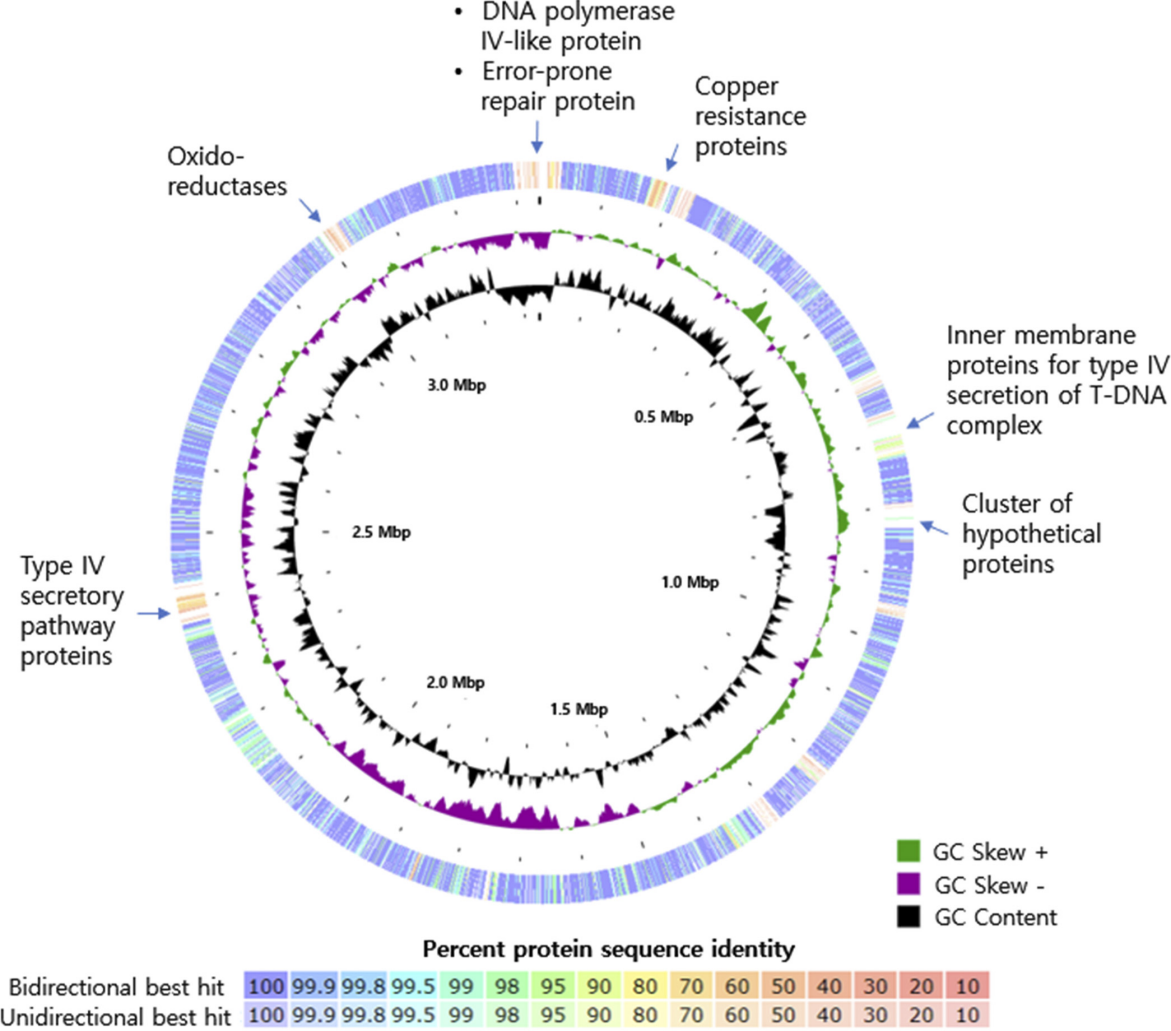
Genome comparison of TT17 to KEME 9005-016^T^ conducted using Rapid Annotations using Subsystems Technology (RAST) (outside track) and GC skew and GC content plots of TT17 (inside). The track of TT17 represents pairwise BLAST comparison between the open reading frames (ORFs) in TT17 genome against those in KEME 9005-016^T^, with percentages of identity represented with different colors shown in the legend.

Table 1 represents the general features of the genomes of TT17 and KEME 9005-016^T^. The assembly report provided by NCBI (ASM2357356v1) showed that TT17 is a type category of B. albigilva. The pairwise BLAST comparison between the open reading frames (ORFs) in the two genomes using RAST serve showed overall very high similarities. Still, some clusters with lower similarities and nonmatches are scattered as found in the TT17 genome (Fig. 1).

**TABLE 1:** General features of the B. albigilva genome sequencing and assembly

Feature	TT17	KEME 9005-016^T^
Isolation	Human skin	Soil
Sequencing analysis		
Illumina		
No. of reads	37,675,580	—^*a*^
Total read length (Mbp)	5,689	
Nanopore		
No. of reads	313,628	671,789
Total read length (Gbp)	3.08	2.40
Mean read length (SD) (bp)	9,834 (11,527)	3,516 (7,376)
*N*_50_ (bp)	18,868	10,349
Assembly analysis		
No. of contigs	1	1
Circularity	Circular	Linear
Size (bp)	3,336,758	3,214,510
GC content (%)	68.90	69.30
Genome coverage (×)	924.0	241.7
No. of protein coding genes	3,245	2,889
No. of rRNA genes (5S, 16S, and 23S)	2	2
No. of tRNA genes	49	49
No. of ncRNA genes^*b*^	3	3
Estimated completeness (%)	100.00	95.67
Estimated contamination (%)	0.11	0.32

a —, not sequenced.

*^b^* ncRNA, noncoding RNA.

### Data availability.

The assembled genome sequences of B. albigilva TT7 and KEME 9005-016^T^ were deposited in GenBank under the accession numbers CP097649 and CP097298, respectively. Raw sequence data used for assembly were deposited in GenBank under SRA accession numbers SRX15388289 (Illumina Nova seq) and SRX15388290 (MinIon seq) for strain TT17 and SRX14829765 (MinIon seq) for KEME 9005-016^T^.
